# Young People’s Intentional and Unintentional Encounters with Internet Pornography in Australia

**DOI:** 10.1007/s10508-025-03109-2

**Published:** 2025-03-17

**Authors:** Savannah Minihan, Melanie Burton, Katherine Giunta, Laureen Villegas, Mariesa Nicholas

**Affiliations:** eSafety Commissioner, Sydney, 6009 Australia

**Keywords:** Internet pornography, Adolescence, Prevalence

## Abstract

Amidst the increased online engagement of adolescents, a growing global body of literature has examined adolescents’ encounters with internet pornography (IP). Though prevalence rates vary across studies, collectively the literature suggests that most adolescents have encountered IP. However, there is a lack of recent Australian research regarding adolescents’ encounters with IP, in particular research that distinguishes between intentional and unintentional encounters. The present study provides updated evidence on the prevalence of intentional and unintentional encounters with IP among Australian adolescents (*n* = 1004, 16–18 years) and examines sociodemographic characteristics associated with encounters. Approximately 75% of adolescents reported having ever encountered IP, with 40% of adolescents first encountering it under the age of 13 and accidentally. Most adolescents (63%) indicated that they encountered IP monthly or more often. Older adolescents, boys, sexually diverse adolescents, and disabled young people, were more likely to have ever encountered IP. Sexually diverse adolescents, young people with disability, and those who spoke a language other than English at home were also significantly more likely to first encounter IP at a younger age. Girls were more likely than boys to report first encountering IP unintentionally and were also more likely to report subsequent accidental encounters with IP. Conversely, boys were more likely to report encountering IP intentionally and recurrently. Finally, participants who reported being under 13 years of age when they first encountered IP were more likely to report recurrent and intentional encounters with IP. Our findings indicate that IP is highly visible in the online lives of Australian adolescents, with many encounters likely occurring unintentionally. Evidently, more stringent measures are needed on online platforms to prevent adolescents from unintentionally encountering IP.

**Supplementary Information:**

The online version contains supplementary material available at 10.1007/s10508-025-03109-2.

## Introduction

Amidst the rise of unmonitored, online digital technologies, the rapid evolution of social media, and the increased online engagement of adolescents (Lim et al., 2017; Martellozzo et al., 2020; McKee, 2010), a growing body of literature has examined adolescents’ encounters with internet pornography (IP) (Hornor, 2020; Jhe et al., 2023; Peter & Valkenberg, 2016; Svedin et al., 2022). There is a lack of recent Australian research, however, into adolescents’ encounters with IP, particularly research that distinguishes between intentional and unintentional encounters. The present study provides updated evidence on the prevalence of intentional and unintentional encounters with IP among Australian adolescents and examines sociodemographic characteristics associated with encounters.

The prevalence of adolescent encounters with IP varies between studies, influenced by the location and age of participants, definitions of IP used, research design, and the rapid change in ways in which adolescents are engaging with technology (Fisher et al., 2013; Goldstein, 2018; Peter & Valkenburg, 2016). Despite these varying prevalence rates, a growing number of international studies have indicated that most adolescents have encountered IP (e.g., Bőthe et al., 2020; Harsey et al., 2021; Henry & Talbot, 2019; Lobe et al., 2011; Noll et al., 2022; Robb & Mann, 2023; Thurman & Obster, 2021; Wright et al., 2020). Australian studies have also shown that encounters with IP are common among adolescents (e.g., Allen & Laborde, 2022; Crabbe et al., 2024; Flood, 2007; Lim et al., 2017; Power et al., 2022; Warren & Swarmi, 2018), with recent research suggesting that 85% of Australian adolescents have encountered IP (Lim et al., 2017; Power et al., 2022).

Existing studies suggest that several sociodemographic characteristics are associated with whether young people have ever encountered IP, the age at which they first encounter IP, and the frequency with which they subsequently do so. For example, some studies have shown that encounters with IP are more prevalent among Australian boys compared with girls (Allen & Laborde, 2022; Crabbe et al., 2024; Flood, 2007; Lim et al., 2017; Power et al., 2022). Several studies have also found that Australian boys first encounter IP at a younger age and more frequently compared with girls (Bernstein et al., 2023; Crabbe et al., 2024; Lim et al., 2017). In addition, emerging evidence suggests that sexually diverse Australian adolescents may be more likely to encounter IP (Power et al., 2022), to encounter it more frequently, and from a younger age (Lim et al., 2017). There are other sociodemographic characteristics that may be associated with encounters with IP but that remain under-explored, such as lived experience of disability and linguistic diversity. Indeed, there is some evidence to suggest that young people with disability and those from culturally and racially marginalized backgrounds lack adequate and representative sex and relationships education (Commissioner for Children & Young People, 2021; McDaniels & Fleming, 2016; Michielsen & Brockschmidt, 2021).^1^ If a young person is unable to access comprehensive education through mainstream means (i.e., school), they may turn to alternative means, such as IP, to augment their education.

### Intentional Encounters with Internet Pornography

When examining adolescents’ encounters with IP, it is important to distinguish between unintentional and intentional encounters (Flood, 2007). Intentional encounters involve a deliberate search for IP (Flood, 2007; Tsaliki, 2011), which may occur out of curiosity, for pleasure, or for entertainment (Healy-Cullen et al., 2022; Henry & Talbot, 2019; Power et al., 2022). International studies have found that many adolescents intentionally encounter IP (Chen et al., 2013; Hardy et al., 2013; Henry & Talbot, 2019; Martellozzo et al., 2016, 2020; Noll et al., 2022; Robb & Mann, 2023; Ybarra et al., 2011), with similar findings reported in Australian studies. For example, Bernstein et al., (2022, 2023) reported that approximately 80% of Australian undergraduate students who had encountered IP had actively searched for IP, and 60% first encountered it intentionally. Further, Biswas et al. (2021) reported that 40% of Australian 18–19-year-olds had intentionally engaged with pornography in their lifetime.

The literature suggests that sociodemographic characteristics may be associated with intentional encounters with IP among young people. International studies have shown that boys are more likely to intentionally encounter IP than girls (Camilleri et al., 2021; Henry & Talbot, 2019; Martellozzo et al., 2016; Robb & Mann, 2023; Rothman et al., 2015) and that sexually diverse adolescents are more likely to intentionally encounter IP than straight adolescents (Bőthe et al., 2019, 2020; Luder et al., 2011). Consistent with international findings, Biswas et al. (2021) found that more boys (62%) than girls (22%) had intentionally engaged with pornography. Similarly, Waren and Swarmi (2018) reported that 63% of boys and 18% of girls aged 16–17 years had encountered pornography intentionally in the past 12-months. To our knowledge, there has been no research focusing on trans and gender-diverse young people’s encounters with IP. While it is possible to assume that the existing literature on boy’s and girl’s encounters with pornography is inclusive of both cis and trans boys and girls, more data are needed on the experiences of trans and gender-diverse young people in relation to IP. There is also a lack of Australian research that has examined differences between intentional encounters with IP among straight and sexually diverse adolescents.

### Unintentional Encounters with Internet Pornography

Unintentional encounters with IP occur when adolescents inadvertently or accidentally encounter IP. Due to the nature of such encounters, adolescents are unlikely to be prepared for what they see (Massey et al., 2021). Unintentional encounters can occur through accidental viewing, including via pop-up images and advertisements (Ševcíkova et al., 2014), typing in the wrong search terms or website address (Flood, 2007), or by clicking on unknown links (Thornburgh & Lin, 2002). Unintentional encounters can also occur via the deliberate or intrusive actions of others, including unsolicited messages, emails, and links to material (Chen et al., 2013; Flood, 2007).

There is a growing body of international literature showing that many adolescents unintentionally encounter IP (e.g., Hardy et al., 2013; Harsey et al., 2021; Healy-Cullen et al., 2022; Henry & Talbot, 2019; Martellozzo et al., 2016; Mitchell et al., 2007; Peter & Valkenburg, 2016; Robb & Mann, 2023; Wolak et al., 2007), through a variety of means (Robb & Mann, 2023). In a US survey, one in two adolescents had unintentionally encountered IP accidentally via clicking a link, a search engine result, an online ad, or on social media. Further, 23% had unintentionally seen IP as a result of a friend or classmate they knew offline having shown it to them, and 12% when a friend or classmate they only knew online showed it to them (Robb & Mann 2023). Consistent with this, Bernstein et al. (2023) found that approximately 40% of Australian undergraduate students first encountered IP unintentionally. In addition, qualitative research with Australian young people has highlighted the pervasive nature of IP via pop-up advertisements that redirect to porn sites and the frequency of unintentional encounters with IP via social networking sites (Lewis et al., 2018; Walker et al., 2015). There is some international evidence to suggest that adolescent girls may be more likely to first encounter IP unintentionally compared with boys (Camilleri et al., 2021; Henry & Talbot, 2019). While there is a lack of recent empirical evidence in the Australian context (but see Crabbe et al., 2024; Our Watch, 2020), an early Australian study indicated that adolescent boys may be more likely to have ever unintentionally encountered IP, compared with adolescent girls (Flood, 2007).

Collectively, the available literature indicates that both intentional and unintentional encounters with IP are prevalent among adolescents, with many adolescents first encountering IP at a young age. However, there is a lack of recent Australian research regarding adolescents’ encounters with IP, in particular research that distinguishes between intentional and unintentional encounters. The evidence also suggests that there may be certain sociodemographic characteristics associated with encounters with IP, including gender and sexuality; however, other potentially relevant factors, such as lived experience of disability and linguistic diversity, remain under-explored. In this context, the present study had two aims: First, to provide an updated prevalence estimate for encounters with IP among Australian adolescents. The present study aimed to build on prior research on the prevalence of encounters with IP by (1) differentiating between intentional and unintentional encounters; (2) differentiating between lifetime and first encounters; and (3) examining frequency of encounters. The second aim was to examine the relationship between encounters with IP and sociodemographic characteristics such as current age, gender, sexuality, lived experience of disability, and linguistic diversity. Given some research showing that younger age of first encounter with IP is associated with more frequent IP viewing (Bernstein et al., 2023; Office of Film and Literature Classification, 2018), we also explored the relationship between age of first encounter with IP and frequency and means of encounters with IP.

## Method

Data were collected through a cross-sectional survey as part of a broader mixed methods study about Australian adolescents’ perspectives on IP and attitudes toward age assurance. For the full description of survey items, please contact the corresponding author.

eSafety understands the impact of researchers’ intersecting experiences of power and marginalization on our research and analysis. The team that authored this paper is made up of cis-gender women of European and Asian heritage. Identities represented in the team include queer women and people with disability. Our team has expertise in quantitative methodologies, online harms and safety, and the lived experiences of young people.

### Participants

A non-probability-based online panel provider (Octopus Group) was used to recruit participants, with quotas placed on gender, age, location, linguistic diversity, sexual orientation, and lived experience of disability. Participants were recruited directly, rather than via their parents or carers. Recruiting via parental or carer referral could prevent the participation of many adolescents, given the sensitive nature of the research. Adolescents in out-of-home care, those who are not close with their parents or carers, and those who do not feel comfortable discussing subjects such as IP with their parents, stood to be excluded from this study if contact via parents was the main form of recruitment. In accordance with the National Health and Medical Research Council Statement on Ethical Conduct in Human Research (2018), only mature minors were included as participants. The survey was conducted Australia-wide. A total of 1004 adolescents, aged 16–18 years, participated. See Table 1 for sociodemographic characteristics of participants.

**Table 1:** Participant sociodemographic characteristics

	%	*n*
*Gender*		
Boys	36.1	362
Girls	61.5	617
Non-binary and gender diverse	2.2	22
Prefer not to answer	0.3	3
*Age*		
16 years	33.7	338
17 years	33.2	333
18 years	33.2	333
*Country of birth*		
Australia	82.8	831
Outside Australia	16.8	169
Prefer not to answer	0.4	4
*Speak language other than English at home*		
Yes	24.6	247
No	74.5	748
Unsure	0.4	4
Prefer not to answer	0.5	5
*Sexuality*		
Sexually diverse	21.8	219
Straight	76.8	771
Don’t know	0.7	7
Prefer not to answer	0.7	7
*Lived experience of disability*		
Yes	22.7	228
No	74.2	745
Prefer not to answer	3.1	31
*First nations*		
Yes	3.1	31
No	95.9	963
Unsure	0.5	5
Prefer not to answer	0.5	5

Boys includes 356 cis and 6 transgender boys; girls includes 614 cis and 3 transgender girls; non-binary and gender diverse includes 15 non-binary adolescents, four who are unsure/questioning, and three who use a different term for their gender than those listed in the survey. Sexually diverse includes participants who identified their sexuality as: gay, lesbian, bisexual, queer, asexual, or unsure/questioning and those who used a different term

### Measures

#### Encounters with Internet Pornography

Participants were asked a series of questions regarding their encounters with IP. IP was defined as: “Textual, visual and audio-visual sexually explicit material that is primarily intended to sexually arouse the audience. This can include representations of images of nudity or semi-nudity, implied sexual activity and actual sexual activity that is uploaded, accessed and shared via online platforms. This does not include the sending or receiving of nudes or nude selfies, also known as sexting.”

Participants were first asked whether they have ever seen IP, with response options, “Yes,” “No,” “Unsure,” and “Prefer not to answer.” Participants who responded “Yes” were asked a series of follow-up questions about their first and subsequent encounters with IP. Specifically, participants were asked: how old they were when they first saw IP; how they first saw IP; how often they have seen IP; and how they see IP. For age of first encounter with IP, participants could select one response from 12 options, ranging from “under 8 years” to “18 years.” For means of first encounter with IP, participants could select one response from 10 options: “I searched for it online”; “Someone sent it to me in a private message”; “Someone showed it to me on their device”; “Someone shared it in a group chat”; “It appeared when I searched online for something else”; “It appeared in ads while I was on social media”; “It appeared in ads while I was online e.g., on gaming sites, news sites”; “Other”; “Prefer not to answer.” For frequency of encounters with IP, participants could select one response from seven options: “Once or twice”; “Once a month”; “Once a week”; “Daily”; “Several times a day”; “Unsure”; “Prefer not to answer.” Finally, for means of encounters with IP, participants could select multiple responses from six options: “I specifically looked for this content”; “I asked someone to send it to me/show it to me”; “Someone sent it to me without asking me”; “By accident”; “Other”; “Prefer not to answer.”

### Procedure

To ensure they could make an informed decision about study participation, and to ascertain their suitability to take part as mature minors and their capacity to provide informed consent, participants were first asked their age, followed by two multiple choice questions (Friedman et al., 2016; Mackenzie et al., 2021). The questions referred to information from the participant information sheet. Participants were given two attempts to answer correctly. Participants who answered both questions correctly were eligible to participate in the study. Following provision of consent, participants commenced the survey. The survey took approximately 15 min and consisted of 40 multiple choice close-ended questions.

### Data Analysis

Prior to commencing data analysis, data on the variables of interest were aggregated. Specifically, given that age of first encounter with IP was not measured on a linear scale, as well as low cell sizes across several response options, this variable was dichotomized based on a median split. The dichotomized variable compared those who were under 13 when they first saw IP, to those who were 13 or older. Given our interest in distinguishing between intentional and unintentional encounters with IP, we also aggregated responses on the variables assessing how participants encountered IP. Specifically, for first encounters with IP, responses were collapsed to compare intentional (i.e., “I searched for it online”) with accidental encounters (i.e., “It appeared in my social media feed”; “It appeared when I searched online for something else”; “It appeared in ads while I was on social media”; “It appeared in ads while I was online e.g., on gaming sites, news sites”), and encounters which involved being shown IP by someone else (i.e., “Someone sent it to me in a private message”; “Someone showed it to me on their device’; “Someone shared it in a group chat”). Because the variable assessing encounters with IP, in general, allowed for multiple responses, several dummy variables were computed. Specifically, we computed a dummy variable to index: intentional encounters (i.e., “I specifically looked for this content” and “I asked someone to send it to me/show it to me”); accidental encounters (i.e., “By accident”); and non-consensual encounters (i.e., “Someone sent it to me without asking me”). Finally, the variable assessing frequency of encounters with IP was dichotomized to compare recurrent (i.e., “Once a month”; “Once a week”; ‘Daily’; “Several times a day”) with infrequent encounters (i.e., “Once or twice”). A series of logistic and multinomial logistic regression models were then conducted to investigate sociodemographic characteristics associated with having ever encountered IP, as well as with characteristics of first and subsequent encounters with IP.

## Results

For descriptive statistics of study variables, see Supplementary Table 1.

### Sociodemographic Characteristics Associated with Having Ever Encountered Internet Pornography

Most adolescents (74.8%, *n* = 751) surveyed had encountered IP. This was the case for 79.3% (*n* = 287) of boys, 72.1% (*n* = 445) of girls. In addition, 68.6% (*n* = 232) of 16-year-olds, 74.8% (*n* = 249) of 17-year-olds and 81.1% (*n* = 270) of 18-year-olds had encountered IP.

A logistic regression model was specified to explore sociodemographic characteristics associated with having ever encountered IP. The model demonstrated good fit as indicated by the Hosmer and Lemeshow test (*χ*^2^(7) = 6.27, *p* = .508), and the model was statistically significant, *χ*^*2*^(6) = 56.47, *p* < .001 (Nagelkerke *R*^*2*^ = .10). Analyses showed that having ever encountered IP was significantly associated with current age, gender identity, sexuality, and lived experience of disability (Table 2). Compared with 16-year-olds, 18-year-olds were over twice as likely to have encountered IP, while 17-year-olds were 1.5 times as likely. Boys were over twice as likely to have ever encountered IP compared with girls and sexually diverse adolescents were over 3 times as likely to have ever encountered IP compared with straight adolescents. Adolescents with disability were almost twice as likely to have ever encountered IP, compared with those without disability (Table 2).

**Table 2:** Sociodemographic characteristics associated with having ever encountered internet pornography

Predictor	B	SE	OR	95% CI for OR	*p*
*Current age*					
16 years	Ref	Ref	Ref	Ref	Ref
17 Years	0.42	0.20	1.53	1.03–2.27	.037
18 Years	0.91	0.22	2.49	1.63–3.81	< .001
*Gender identity*					
Boy	Ref	Ref	Ref	Ref	Ref
Girl	− 0.79	0.19	0.46	0.31–0.66	< .001
*Sexuality*					
Straight	Ref	Ref	Ref	Ref	Ref
Sexually diverse	1.11	0.27	3.02	1.77–5.16	< .001
*Language other than English*					
No	Ref	Ref	Ref	Ref	Ref
Yes	0.05	0.20	1.05	0.70–1.56	.817
*Lived experience of disability*					
No	Ref	Ref	Ref	Ref	Ref
Yes	0.56	0.23	1.75	1.11–2.75	.017

For the dependent variable, never having encountered IP is the reference category. Non-binary and gender-diverse individuals and those who responded “prefer not to answer,” “other,” or “unsure” to any variable were excluded from analyses. The model therefore included *n* = 885

### Sociodemographic Characteristics Associated with Age of First Encounter with Internet Pornography

Of those participants who had ever encountered IP, almost 40% (*n* = 294) of participants reported being under 13 when they first encountered IP. A logistic regression model was specified to explore sociodemographic characteristics associated with age of first encounter with IP, with 13 years and over being the reference category. The model fit the data well (*χ*^*2*^(8) = 11.63, *p* = .168) and was statistically significant, *χ*^*2*^(6) = 50.46, *p* < .001 (Nagelkerke *R*^*2*^ = .10). Analyses showed that age of first encounter with IP was significantly associated with sexuality, speaking a language other than English at home, and lived experience of disability, but not with gender identity (Table 3). Specifically, sexually diverse adolescents were more than twice as likely as straight adolescents to report being under 13 when they first encountered IP. Those who spoke a language other than English at home and those with lived experience of disability were almost twice as likely to report being under 13 when they first encountered IP, compared with those who spoke only English at home and those without lived experience of disability, respectively.

**Table 3:** Sociodemographic characteristics associated with age of first encounter with internet pornography

Predictor	B	SE	OR	95% CI for OR	*p*
*Current age*					
16 years	Ref	Ref	Ref	Ref	Ref
17 Years	0.39	0.20	1.47	0.99–2.19	.056
18 Years	− 0.08	0.21	0.92	0.62–1.38	.687
*Gender identity*					
Boy	Ref	Ref	Ref	Ref	Ref
Girl	0.05	0.18	1.05	0.75–1.48	.786
*Sexuality*					
Straight	Ref	Ref	Ref	Ref	Ref
Sexually diverse	0.82	0.19	2.26	1.56–3.28	< .001
*Language other than English*					
No	Ref	Ref	Ref	Ref	Ref
Yes	0.49	0.19	1.63	1.14–2.35	.008
*Lived experience of disability*					
No	Ref	Ref	Ref	Ref	Ref
Yes	0.61	0.19	1.84	1.28–2.65	< .001

For the dependent variable, 13 years and over is the reference category. Participants who had never encountered IP, non-binary and gender-diverse individuals and those who responded “prefer not to answer,” “other,” or “unsure” to any variable were excluded from analyses. The model therefore included *n* = 699

### Sociodemographic Characteristics Associated with Means of First Encounter with Internet Pornography

Almost 40% (*n* = 300) of participants who had ever encountered IP reported that they first encountered it accidentally, compared with 34.4% (*n* = 258) who first encountered IP by being shown or sent it by someone, and 21.6% (*n* = 162) who first encountered IP intentionally. A multinomial logistic regression model was specified to examine sociodemographic characteristics associated with means of first encounter with IP. The model represented a good fit to the data, according to a likelihood ratio test relative to an intercept-only model (*χ*^*2*^(12) = 50.08, *p* < .001). The Nagelkerke *R*^*2*^ statistic was.08. Likelihood ratio tests of individual predictors were significant for gender identity, *χ*^*2*^(2) = 18.83, *p* < .001; speaking a language other than English at home, *χ*^*2*^(2) = 15.43, *p* < .001; and lived experience of disability, *χ*^*2*^(2) = 6.48, *p* = .039. Girls were almost twice as likely as boys to first encounter IP by being sent or shown it by someone, as opposed to searching for it intentionally (Table 4). Girls were also almost three times as likely as boys to first encounter IP accidentally, as opposed to intentionally. Individuals who spoke a language other than English at home were less likely than those who spoke English at home to first encounter IP by being sent or shown it by someone, as opposed to searching for it intentionally. While the overall likelihood ratio test for lived experience of disability was significant, all the individual comparisons were non-significant.

**Table 4:** Sociodemographic characteristics associated with means of first encounter with internet pornography

Predictor	Sent/Shown It					Accidental				
	B	SE	OR	95% CI for OR	*p*	B	SE	OR	95% CI for OR	*p*
*Current age*										
16 Years	Ref	Ref	Ref	Ref	Ref	Ref	Ref	Ref	Ref	Ref
17 Years	− 0.13	0.27	0.88	0.52–1.49	.634	− 0.14	0.27	0.87	0.52–1.48	.616
18 Years	− 0.66	0.26	0.52	0.31–0.86	.012	− 0.38	0.26	0.69	0.41–1.14	.147
*Gender identity*										
Boy	Ref	Ref	Ref	Ref	Ref	Ref	Ref	Ref	Ref	Ref
Girl	0.58	0.22	1.79	1.15–2.75	.010	0.96	0.22	2.61	1.67–4.04	< .001
*Sexuality*										
Straight	Ref	Ref	Ref	Ref	Ref	Ref	Ref	Ref	Ref	Ref
Sexually diverse	− 0.29	0.26	0.75	0.45–1.25	.267	− 0.01	0.25	0.99	0.61–1.60	.961
*Language other than English*										
No	Ref	Ref	Ref	Ref	Ref	Ref	Ref	Ref	Ref	Ref
Yes	− 0.56	0.25	0.57	0.35–0.94	.028	0.28	0.23	1.32	0.84–2.09	.226
*Lived experience of disability*										
No	Ref	Ref	Ref	Ref	Ref	Ref	Ref	Ref	Ref	Ref
Yes	− 0.39	0.25	0.68	0.41–1.11	.125	0.15	0.24	1.16	0.73–1.84	.543

For the dependent variable, intentional is the reference category. Participants who had never encountered IP, non-binary and gender-diverse individuals and those who responded “prefer not to answer,” “other,” or “unsure” to any variable were excluded from analyses. The model therefore included *n* = 673

### Sociodemographic Characteristics Associated with Frequency of Encounters with Internet Pornography

Almost two-thirds (*n* = 476, 63.4%) of participants who had ever encountered IP reported recurrent (i.e., monthly or more) encounters with IP. A logistic regression model was specified to examine sociodemographic characteristics associated with frequency of encounters with IP. The model fit the data well (*χ*^*2*^(8) = 10.00, *p* = .265) and was statistically significant, *χ*^*2*^(7) = 75.39, *p* < .001 (Nagelkerke *R*^*2*^ = .16). Current age was significantly associated with frequency of encounters with IP: 18-year-olds were over twice as likely as 16-year-olds to encounter IP monthly or more, as opposed to only once or twice (Table 5). Conversely, there were no significant differences between 16- and 17-year-olds in frequency of encounters with IP. Gender identity was also significantly associated with frequency of encounters with IP, with boys over twice as likely as girls to encounter IP recurrently. Age of first encounter with IP was also significantly associated with frequency of encounters with IP: Those who first encountered IP when they were under 13 were over 3 times as likely to report recurrent encounters with IP, compared with those who first encountered IP when they were 13 or older.

**Table 5:** Sociodemographic characteristics associated with frequency of encounters with internet pornography

Predictor	B	SE	OR	95% CI for OR	*p*
*Current age*					
16 Years	Ref	Ref	Ref	Ref	Ref
17 Years	0.11	0.23	1.11	0.71–1.75	.649
18 Years	0.92	0.24	2.52	1.56–4.06	< .001
*Gender identity*					
Boy	Ref	Ref	Ref	Ref	Ref
Girl	− 1.03	0.22	0.36	0.23–0.54	< .001
*Sexuality*					
Straight	Ref	Ref	Ref	Ref	Ref
Sexually diverse	0.34	0.24	1.40	0.87–2.25	.165
*Language other than English*					
No	Ref	Ref	Ref	Ref	Ref
Yes	0.34	0.23	1.41	0.89–2.21	.143
*Lived experience of disability*					
No	Ref	Ref	Ref	Ref	Ref
Yes	0.22	0.23	1.25	0.80–1.96	.328
*Age of first encounter*					
13 and over	Ref	Ref	Ref	Ref	Ref
Under 13	1.25	0.22	3.49	2.27–5.36	< .001

For the dependent variable, once or twice is the reference category. Participants who had never encountered IP, non-binary and gender-diverse individuals and those who responded “prefer not to answer,” “other,” or “unsure” to any variable were excluded from analyses. The model therefore included *n* = 624

### Means of Encounters with Internet Pornography

Of those participants who had ever encountered IP, 60.9% (*n* = 457) reported that they encounter IP intentionally. Over half of participants (*n* = 437, 58.2%) also indicated that they accidentally encounter IP, and over a quarter (*n* = 211, 28.1%) indicated that they have encountered IP non-consensually. Three logistic regression models were specified to examine sociodemographic correlates of means of encounters with IP.

In the first model, intentional encounters with IP was included as the dependent variable. The model was statistically significant, *χ*^*2*^(7) = 67.40 *p* < .001, and fit the data well, *χ*^*2*^(8) = 4.38, *p* = .821 (Nagelkerke *R*^*2*^ = .13). Age, gender identity, sexuality, and age of first encounter with IP were significantly associated with intentional encounters with IP (Table 6, Model 1). Specifically, 18-year-olds were over three times as likely to encounter IP intentionally, compared with 16-year-olds, while 17-year-olds were over 1.5 times as likely. Boys were over twice as likely to encounter IP intentionally, compared with girls. Adolescents who identified as sexually diverse were more likely than straight adolescents to intentionally encounter IP. Those who first saw IP when they were under 13 years were over twice as likely to report encountering IP intentionally, compared with those who first saw IP when they were 13 years or older.

**Table 6:** Sociodemographic characteristics associated with means of encounters with internet pornography generally

Predictor	Intentional					Accidental					Non-consensual				
	B	SE	OR	95% CI for OR	*p*	B	SE	OR	95% CI for OR	*p*	B	SE	OR	95% CI for OR	*p*
*Current age*															
16 years	Ref	Ref	Ref	Ref	Ref	Ref	Ref	Ref	Ref	Ref	Ref	Ref	Ref	Ref	Ref
17 Years	0.47	0.21	1.61	1.07–2.42	.023	− 0.45	0.21	0.64	0.43–0.96	.032	− 0.49	0.22	0.61	0.40–0.94	.023
18 Years	1.13	0.22	3.09	2.02–4.74	< .001	− 0.65	0.21	0.52	0.35–0.78	.002	− 0.50	0.21	0.61	0.40–0.92	.019
*Gender identity*															
Boy	Ref	Ref	Ref	Ref	Ref	Ref	Ref	Ref	Ref	Ref	Ref	Ref	Ref	Ref	Ref
Girl	− 0.95	0.19	0.39	0.27–0.57	< .001	0.72	0.18	2.05	1.45–2.88	< .001	0.34	0.19	1.40	0.97–2.04	.075
*Sexuality*															
Straight	Ref	Ref	Ref	Ref	Ref	Ref	Ref	Ref	Ref	Ref	Ref	Ref	Ref	Ref	Ref
Sexually diverse	0.44	0.21	1.55	1.02–2.35	.043	− 0.35	0.20	0.71	0.48–1.05	.083	0.14	0.21	1.15	0.77–1.72	.504
*Language other than English*															
No	Ref	Ref	Ref	Ref	Ref	Ref	Ref	Ref	Ref	Ref	Ref	Ref	Ref	Ref	Ref
Yes	0.07	0.20	1.07	0.72–1.59	.748	− 0.21	0.19	0.81	0.56–1.18	.275	− 0.71	0.23	0.49	0.32–0.77	.002
*Lived experience of disability*															
No	Ref	Ref	Ref	Ref	Ref	Ref	Ref	Ref	Ref	Ref	Ref	Ref	Ref	Ref	Ref
Yes	− 0.08	0.20	0.93	0.63–1.37	.707	0.43	0.20	1.54	1.05–2.26	.028	0.22	0.20	1.25	0.84–1.84	.269
*Age of first encounter*															
13 and over	Ref	Ref	Ref	Ref	Ref	Ref	Ref	Ref	Ref	Ref	Ref	Ref	Ref	Ref	Ref
Under 13	0.84	0.19	2.32	1.61–3.35	< .001	0.27	0.17	1.31	0.93–1.84	.122	0.54	0.18	1.71	1.20–2.45	.003

For the dependent variables, no (i.e., no intentional/accidental/non-consensual encounters) is the reference category. Participants who had never encountered IP, non-binary and gender-diverse individuals and those who responded “prefer not to answer,” “other,” or “unsure” to any variable were excluded from analyses. The models therefore included *n* = 672

In the second model, accidental encounters with IP was included as the dependent variable. The model was statistically significant, *χ*^*2*^(7) = 35.23, *p* < .001, and provided an adequate fit to the data, *χ*^*2*^(8) = 17.10, *p* = .029 (Nagelkerke *R*^*2*^ = .07). Age, gender identity, and lived experience of disability were significantly associated with accidental encounters with IP (Table 6, Model 2). Both 18- and 17-year-olds were less likely than 16-year-olds to report accidentally encountering IP. Girls were over twice as likely as boys to report encountering IP accidentally, and those with lived experience of disability were more likely than those without to report accidentally encountering IP.

In the third model, non-consensual encounters with IP was included as the dependent variable. The model was statistically significant, *χ*^*2*^(7) = 34.06, *p* < .001, and fit the data well, *χ*^*2*^(8) = 6.67, *p* = .573 (Nagelkerke *R*^*2*^ = .07). Age, speaking a language other than English at home, and age of first encounter with IP were significantly associated with non-consensual encounters with IP (Table 6, Model 3). Those participants who spoke a language other than English at home were significantly less likely than those who only spoke English at home to report non-consensual encounters with IP, as were those who were 13 years or older, compared with under 13, when they first encountered IP.

## Discussion

The present study examined the prevalence and sociodemographic correlates of Australian adolescents’ intentional and unintentional encounters with IP. Overall, we found that most adolescents surveyed had encountered IP. Although comparing our prevalence rates with previous studies is challenging given differences in operationalization of IP, different time periods between studies, and different age groups examined, our findings are consistent with prior Australian and international research, suggesting that most adolescents globally have encountered IP (Lim et al., 2017; Power et al., 2022; Robb & Mann, 2023; Thurman & Obster, 2021). Our findings also indicated that various sociodemographic characteristics were associated with the nature of encounters with IP.

### The Prevalence of Intentional and Unintentional Encounters with Internet Pornography

Australian and international studies have found that many adolescents intentionally encounter IP (e.g., Chen et al., 2013; Hardy et al., 2013; Henry & Talbot, 2019; Martellozzo et al., 2016, 2020; Noll et al., 2022; Robb & Mann, 2023; Ybarra et al., 2011). Consistent with this, we found that three in five 16–18-year-old adolescents who had encountered IP, had ever intentionally encountered IP, and one in five first encountered IP intentionally. However, although comparisons to other studies should be made with caution, these findings were lower than those observed in recent studies by Bernstein et al., (2022, 2023), who reported that four in five Australian 17–25-year-olds who had encountered IP, subsequently actively searched for it, and three in five first encountered it intentionally. While this may reflect differences in the methodology, it may also indicate that while some early adolescents encounter IP intentionally, an increasing proportion of young people may intentionally seek out IP as they progress through adolescence (Martellozzo et al., 2016) and young adulthood. Consistent with this hypothesis, we also found that both 17- and 18-year-olds were significantly more likely to report encountering IP intentionally, compared with 16-year-olds.

Similar to international research showing high rates of unintentional encounters with IP among adolescents (Hardy et al., 2013; Harsey et al., 2021; Healy-Cullen et al., 2022; Henry & Talbot, 2019; Martellozzo et al., 2016; Mitchell et al., 2007; Peter & Valkenburg, 2016; Robb & Mann, 2023; Wolak et al., 2007), we also found that of 16–18-year-old adolescents who had encountered IP, the majority had also unintentionally encountered IP. Most adolescents had first encountered IP unintentionally, with 40% first encountering it accidentally, and just over a third being shown or sent it by someone.^2^ These findings were higher than those reported by Bernstein et al. (2023), who found that 40% of Australian 17–25-year-olds first encountered IP unintentionally. This likely reflects the inclusion of young adults in the sample, suggesting that adolescents may be more likely to first encounter IP unintentionally compared with those who first encounter IP in early adulthood. In addition, we found that 17- and 18-year-olds were less likely than 16-year-olds to report accidentally or non-consensually encountering IP in general. Future research could help to identify factors that may increase the risk and those that may protect younger adolescents from encountering IP unintentionally, including digital literacy skills and patterns of use of social media platforms.

### The Age of First Encounters and Frequency of Encounters with Internet Pornography

Our findings suggest that many Australian adolescents likely first encounter IP at a young age and subsequently encounter it regularly. In our study, two in five 16–18-year-olds who had seen IP, first encountered it before they were 13, and almost two in three adolescents reported recurrent (i.e., monthly or more) encounters with IP. Our findings also indicated that adolescents who reported first encountering IP at a younger age were more likely to report subsequent intentional encounters and consistent with Bernstein et al. (2023), we found that first encountering IP at a younger age was associated with recurrent subsequent encounters with IP. Once an adolescent has encountered IP, the likelihood of their encountering it again, even unintentionally, may increase. This might be for a variety of reasons, which further research could help to illuminate. Reasons could include increased curiosity and the ways in which search and social media algorithms often rank content, where content similar to that which the user had viewed or engaged with in the past is ranked higher than other content.

### Relationship Between Encounters with Internet Pornography and Gender Identity

Our findings indicated that 16–18-year-old boys were more likely to have encountered IP compared with adolescent girls, consistent with prior Australian (e.g., Allen & Laborde, 2022; Crabbe et al., 2024; Flood, 2007; Lim et al., 2017; Power et al., 2022) and international research (e.g., Bőthe et al., 2020; Donevan et al., 2022; Robb & Mann, 2023; Stanley et al., 2018). In contrast to previous findings (Bernstein et al., 2023; Lim et al., 2017), however, our results showed that Australian boys and girls first encounter IP at a similar age. However, we found that their pathways to first and subsequent encounters with IP often differ. Girls were more likely than boys to first encounter IP accidentally and to subsequently accidentally encounter IP. This finding was partly consistent with a recent Australian study (Crabbe et al., 2024) and international evidence (Camilleri et al., 2021; Henry & Talbot, 2019) that adolescent girls may be more likely to first encounter IP unintentionally compared with boys. Recent international and domestic studies have found that compared with boys, girls are spending more time on social media platforms where unintentional encounters with IP often occur (eSafety Commissioner, 2022; Leonhardt, & Overa, 2021; Nagata et al., 2022). It may be that the more time that is spent on social media platforms, the more opportunities there are for unintentional encounters with IP to occur.

In comparison, adolescent boys were more likely than girls to encounter IP intentionally and recurrently. Compared with adolescent girls, boys may be more likely to encounter IP out of curiosity, for sexual arousal, and entertainment (Abiala & Hernwall, 2013; Arrington-Sanders et al., 2015; Lofgren-Mårtenson & Månsson, 2010; Rothman et al., 2015), resulting in more intentional encounters with IP. While our data here relates to both trans and cis girls and boys, our sample did not contain enough trans boys and girls to report reliable findings about these young people alone. Similarly, our sample did not contain enough non-binary and gender-diverse young people to report findings for this group.

### Relationship Between Encounters with Internet Pornography and Sexuality

We found that sexually diverse adolescents were more likely to have ever encountered IP, to have been under 13 when they first encountered IP and to encounter it intentionally. These findings are partly consistent with Lim et al. (2017), who reported increased pornography viewing among sexually diverse young people and a younger age of first encounter with IP among sexually diverse women. Further, the findings are in line with Australian and international findings that sexually diverse adolescents are more likely to intentionally encounter IP than straight adolescents (Biswas et al. 2021, Bőthe et al., 2019, 2020; Luder et al., 2011; Waren & Swarmi, 2018). This finding may in part reflect a lack of representation in mainstream culture and education of diverse sexualities, and the inconsistent delivery of sexually diverse content in Australian adolescents’ sex and relationships education, which may lead some sexually diverse adolescents to seek out information via pornography (Bőthe et al., 2019; Bradford et al., 2019; British Board of Film Classification, 2020; Ezer et al., 2020; Fisher et al., 2019; Jones & Hillier, 2012; Litsou et al., 2021).

### Relationship Between Encounters with Internet Pornography and Disability

Adolescents with disability were more likely to have ever encountered IP and to have been under the age of 13 when they first did so. To our knowledge, this is the first Australian study to demonstrate an association between disability and IP encounters and there is a lack of international research exploring this association. Research has indicated that there are many barriers to adolescents with disability accessing comprehensive sex and relationships education (McDaniels & Fleming, 2016; Michielsen & Brockschmidt, 2021). Adolescents with disability may find their sex and relationships education provided offline lacking in representation of their experiences or in addressing their needs, or generally lacking from their education. For some adolescents with disability, IP may be perceived as a resource that fills gaps in their sex and relationships education.

However, we found that adolescents with disability were also more likely to have encountered IP accidentally. Research has indicated that social media plays a key role in fostering social interactions and support for adolescents with disability (Haller, 2010; Ellis & Kent, 2016; Morahan-Martin & Schumacher, 2003). This may lead to some adolescents with disability spending more time on social media compared with those without disability. Indeed, some research has found that adolescents with disability spend more time online than adolescents without disability (Kowalski & Toth, 2018). Further research could help to illuminate whether there is a relationship between time spent online and encounters with IP in adolescents with disability.

### Relationship Between Encounters with Internet Pornography and Speaking a Language Other Than English at Home

Adolescents who spoke a language other than English at home were more likely to first encounter IP under the age of 13. There is some evidence to suggest that many adolescents from culturally and racially marginalized backgrounds lack access to adequate sex and relationships education. In a survey of South Australian young people aged 12–22-years, one in four culturally and linguistically diverse young people were taught about relationships and sexual health as part of their religious education (Commissioner for Children & Young People, 2021). Having religious education as the single source of information about these subjects may mean that young people may perceive gaps in their sex and relationships education, which they may attempt to fill by watching IP.

### Implications of Our Study

Our research emphasizes the importance of online services having strong, effective strategies to prevent adolescents from unintentionally encountering IP, as well as effective reporting mechanisms allowing young people to report unintentional encounters with IP. Search engines and messaging apps can play an important gatekeeper role in reducing adolescents’ unintentional access to this content, for example, through default safe search filters, warning messages and the blurring of explicit content. Moreover, the online industry should continuously improve tools and practices to prevent unintentional encounters with IP and should raise awareness of existing measures that are available to adolescents and their parents/caregivers for application at various levels, including device-level, network-level, browser-level, or account-level restrictions. We also found that adolescents reported encountering IP as a result of someone sending it to them, often without their permission. This indicates that adolescents need robust education around consent and age-inappropriate content, particularly around sending unsolicited sexual images to others, and around the emotional and legal implications that this could have for themselves and others.

Further, we found that it was more common for adolescents with disability and sexually diverse adolescents to encounter IP, and at a younger age. It was also more common for linguistically diverse adolescents to encounter IP at a younger age. This indicates that for some adolescents, IP may be perceived as an important source of information about sex and relationships, providing more diverse representation and an ease of access to information that they may not receive in formal education settings. All adolescents should have access to comprehensive respectful relationships and sex education that addresses their unique needs.

While this research highlighted the high prevalence of unintentional encounters with IP among adolescents surveyed, it did not examine the impacts that such encounters have on adolescents. Future research should examine the short- and longer-term impacts of unintentional encounters with IP on adolescents and protective factors that may mitigate any negative impacts. In addition, future research would benefit from examination of factors that increase the risk of unintentional encounters and those that are protective against this risk.

### Limitations of this Research

Our results must be interpreted within the context of the study’s limitations. First, the technologies and platforms enabling IP are in a constant state of change, as are the online habits of young people. Research on IP encounters must be conducted regularly, to ensure that findings are relevant and reliable. The cross-sectional nature of our study also precludes any causal or directional inferences. It is possible that factors not examined here contributed to the results. Moreover, the study collected self-reported, retrospective information, which is subject to recall and self-presentation bias. The level of accuracy with which adolescents remembered how and when they first encountered IP is unknown, as is the extent to which a reticence to report experiences of some encounters with IP influenced the responses provided. For example, even though the survey was anonymous, some adolescents may have felt more comfortable reporting unintentional than intentional encounters with IP. Additionally, online panels are technically convenience samples, which are not representative of the general population. While best efforts were made to recruit to quotas across a range of sociodemographic characteristics, these quotes were not reached for gender. Results of this study may be subject to a range of biases when compared with results from research using probability-based sampling. Potential biases in our sample may have led to potential biases in the data. Therefore, findings from this study regarding encounters with IP should be considered indicative prevalence estimates. Further research among nationally representative samples is required to substantiate our findings. Finally, the responses of First Nations and trans and gender-diverse young people were not analyzed separately due to the small sample size for each group. Our findings are unable to capture the lived experiences of these groups.

### Conclusion

IP appears to be highly prevalent in the online lives of adolescents. While many adolescents view IP intentionally, many also encounter IP unintentionally, with girls especially susceptible to unintentional encounters. In fact, most adolescents’ first encounters with IP were accidental. Evidently, more stringent measures are needed to prevent adolescents from having unintentional encounters with IP. Future research is also required to determine why some adolescents are more likely than others to encounter IP, and at an earlier age, so that effective prevention initiatives may be designed that target these risk factors and promote the acquisition of modifiable protective factors.

## Supplementary Information

Below is the link to the electronic supplementary material.

Supplementary file1 (DOCX 38 kb)

## Data Availability

The data that support the findings of this study are available on reasonable request from the corresponding author.

## Footnotes

1 Throughout this paper, we use person-first language to describe participants in order to reflect their right to identities beyond their disability. We acknowledge that some young people with disability use identity-first language and that both are valid and meaningful forms of self-identification. As we do not know how individual survey participants self-identify, we cannot follow best practice of using the terms that they use for themselves.

2 This may also include a proportion of respondents who requested IP be sent to them.
