# Supplementary material for: Young People’s Intentional and Unintentional Encounters with Internet Pornography in Australia
Source: Arch Sex Behav. 2025 Mar 17;54(4):1575–88. doi: 10.1007/s10508-025-03109-2 (PMC12011934; doi:10.1007/s10508-025-03109-2)
Supplement: Supplementary file 1 — Supplementary file1 (DOCX 38 kb) [file 10508_2025_3109_MOESM1_ESM.docx]

**Supplementary Table 1**

*Descriptive Characteristics of Young People’s Encounters with Internet Pornography*

|  |  | **Net (%)** | **Current Age (%)** | | | **Gender Identity (%)** | | **Sexuality (%)** | | **Speak language other than English at home (%)** | | **Lived Experience of Disability (%)** | |
| --- | --- | --- | --- | --- | --- | --- | --- | --- | --- | --- | --- | --- | --- |
|  |  | *n*=751 | 16 years (*n*=232) | 17 years (*n*=249) | 18 years (n=270) | Boys (*n*=287) | Girls (*n*=445) | Sexually diverse (*n*=191) | Straight (*n*=553) | Yes (*n*=187) | No (*n*=558) | Yes (*n*=190) | No (*n*=539) |
| **Age of first encounter with IP** | |  |  |  |  |  |  |  |  |  |  |  |  |
|  | **13 years or older - Net** | 60.9 | 63.8 | 53.4 | 65.2 | 63.8 | 59.8 | 45.5 | 66.4 | 52.9 | 63.4 | 47.4 | 65.3 |
|  | 18 years | 1.5 | n/a | n/a | 4.1 | 1.4 | 1.6 | 1.0 | 1.6 | 2.1 | 1.3 | 0.5 | 1.7 |
|  | 17 years | 2.9 | n/a | 1.2 | 7.0 | 1.7 | 3.8 | 1.0 | 3.6 | 2.7 | 3.0 | 2.6 | 3.2 |
|  | 16 years | 9.6 | 6.5 | 9.6 | 12.2 | 7.3 | 11.5 | 7.3 | 10.3 | 6.4 | 10.8 | 8.9 | 10.2 |
|  | 15 years | 13.4 | 18.1 | 8.4 | 14.1 | 14.3 | 13.3 | 6.8 | 15.7 | 10.2 | 14.5 | 8.9 | 15.2 |
|  | 14 years | 18.6 | 20.7 | 19.3 | 16.3 | 17.8 | 19.6 | 16.8 | 19.3 | 18.7 | 18.6 | 14.7 | 19.5 |
|  | 13 years | 14.8 | 18.5 | 14.9 | 11.5 | 21.3 | 10.1 | 12.6 | 15.7 | 12.8 | 15.2 | 11.6 | 15.6 |
|  | **Under 13 years - Net** | 39.1 | 36.2 | 46.6 | 34.8 | 36.2 | 40.2 | 54.5 | 33.6 | 47.1 | 36.6 | 52.6 | 34.7 |
|  | 12 years | 14.9 | 12.1 | 21.3 | 11.5 | 16.0 | 14.6 | 17.3 | 13.9 | 19.3 | 13.4 | 17.4 | 14.1 |
|  | 11 years | 7.2 | 6.0 | 6.8 | 8.5 | 5.9 | 7.6 | 9.9 | 6.1 | 8.0 | 7.0 | 6.8 | 7.4 |
|  | 10 years | 8.5 | 9.9 | 8.4 | 7.4 | 7.0 | 9.0 | 14.1 | 6.5 | 11.2 | 7.7 | 13.2 | 7.1 |
|  | 9 years | 4.3 | 3.9 | 5.6 | 3.3 | 3.8 | 4.3 | 7.9 | 3.1 | 4.8 | 4.1 | 6.3 | 3.3 |
|  | 8 years | 2.4 | 2.2 | 2.4 | 2.6 | 2.1 | 2.7 | 3.1 | 2.2 | 2.7 | 2.3 | 4.2 | 1.9 |
|  | Under 8 years | 1.9 | 2.2 | 2.0 | 1.5 | 1.4 | 2.0 | 2.1 | 1.8 | 1.1 | 2.0 | 4.7 | 0.9 |
| **Means of first encounter with IP** | |  |  |  |  |  |  |  |  |  |  |  |  |
|  | **Shown or sent IP - Net** | 34.4 | 39.2 | 34.9 | 29.6 | 36.6 | 33.9 | 28.3 | 36.5 | 23.0 | 38.5 | 27.4 | 37.1 |
|  | Someone sent it to me in a private message | 6.5 | 9.1 | 7.2 | 3.7 | 5.2 | 7.6 | 6.8 | 6.5 | 3.7 | 7.5 | 4.7 | 6.9 |
|  | Someone showed it to me on their device | 23.4 | 22.4 | 26.1 | 21.9 | 25.8 | 22.7 | 19.9 | 24.6 | 17.1 | 25.8 | 19.5 | 25.2 |
|  | Someone shared it in a group chat | 4.4 | 7.8 | 1.6 | 4.1 | 5.6 | 3.6 | 1.6 | 5.4 | 2.1 | 5.2 | 3.2 | 5.0 |
|  | **Accidentally encountered IP - Net** | 39.9 | 38.4 | 39.0 | 42.2 | 30.7 | 44.7 | 47.1 | 37.4 | 49.2 | 36.6 | 47.9 | 37.1 |
|  | It appeared in my social media feed | 5.2 | 5.2 | 4.4 | 5.9 | 3.8 | 6.3 | 5.8 | 5.1 | 3.7 | 5.6 | 4.2 | 5.8 |
|  | It appeared when I searched online for something else | 13.6 | 13.8 | 15.7 | 11.5 | 12.2 | 14.4 | 18.3 | 11.8 | 18.7 | 11.6 | 20.0 | 11.1 |
|  | It appeared on ads when I was on social media | 5.7 | 4.7 | 4.8 | 7.4 | 3.1 | 7.6 | 5.2 | 6.0 | 5.3 | 5.7 | 5.8 | 5.8 |
|  | It appeared on ads when I was online e.g., gaming sites, news sites | 15.4 | 14.7 | 14.1 | 17.4 | 11.5 | 16.4 | 17.8 | 14.6 | 21.4 | 13.6 | 17.9 | 14.5 |
|  | **Intentionally encountered IP** |  |  |  |  |  |  |  |  |  |  |  |  |
|  | I searched for it online | 21.6 | 19.4 | 20.9 | 24.1 | 28.9 | 17.3 | 20.4 | 22.1 | 23.0 | 21.1 | 21.1 | 21.7 |
| **Frequency of encounters with IP** | |  |  |  |  |  |  |  |  |  |  |  |  |
|  | Once or twice | 25.2 | 30.2 | 26.1 | 20.0 | 19.2 | 29.0 | 20.4 | 26.8 | 18.7 | 27.6 | 22.6 | 26.7 |
|  | **Recurrent encounters - Net** | 63.4 | 57.8 | 62.7 | 68.9 | 70.7 | 58.4 | 70.2 | 61.3 | 70.6 | 61.1 | 68.4 | 61.8 |
|  | Once a month | 27.2 | 22.4 | 28.1 | 30.4 | 17.8 | 33.0 | 28.8 | 26.8 | 27.8 | 26.9 | 27.4 | 26.9 |
|  | Once a week | 23.3 | 23.3 | 22.1 | 24.4 | 27.9 | 20.0 | 28.3 | 21.7 | 26.2 | 22.4 | 28.9 | 21.9 |
|  | Daily | 10.5 | 9.9 | 9.6 | 11.9 | 21.3 | 4.0 | 11.0 | 10.3 | 12.8 | 9.9 | 11.6 | 10.0 |
|  | Several times a day | 2.4 | 2.2 | 2.8 | 2.2 | 3.8 | 1.3 | 2.1 | 2.5 | 3.7 | 2.0 | 0.5 | 3.0 |
| **Means of encounters with IP - general** | |  |  |  |  |  |  |  |  |  |  |  |  |
|  | **Accidentally encountered IP** |  |  |  |  |  |  |  |  |  |  |  |  |
|  | Accidentally encountered IP | 58.2 | 63.8 | 57.0 | 54.4 | 49.1 | 64.0 | 58.6 | 57.7 | 51.9 | 60.2 | 66.8 | 54.9 |
|  | **Non-consensually encountered IP** |  |  |  |  |  |  |  |  |  |  |  |  |
|  | Sent IP without asking | 28.1 | 34.1 | 25.3 | 25.6 | 24.0 | 31.0 | 34.0 | 26.0 | 18.2 | 31.7 | 35.3 | 25.6 |
|  | **Intentionally encountered IP - Net** | 60.9 | 50.9 | 61.8 | 68.5 | 69.0 | 55.1 | 67.5 | 59.0 | 64.2 | 60.0 | 63.2 | 60.7 |
|  | Specifically looked for IP | 58.6 | 48.7 | 59.8 | 65.9 | 67.6 | 52.1 | 67.0 | 56.1 | 63.1 | 57.5 | 61.6 | 58.1 |
|  | Asked someone else to send/show IP to me | 8.9 | 8.2 | 6.8 | 11.5 | 9.8 | 8.8 | 9.4 | 8.9 | 6.4 | 9.7 | 12.1 | 7.6 |

*Note*. Boys includes cis and transgender boys; girls includes cis and transgender girls. Sexually diverse includes participants who identified their sexuality as: gay, lesbian, bisexual, queer, asexual, or unsure/questioning. Column percentages do not always add to 100% as percentages for participants who responded ‘prefer not to answer’, ‘other’, or ‘unsure’ are not reported. Column percentages for ‘Means of encounters with IP – general’ exceed 100% as this was a multi-response variable.
